# Draft Genome Sequence of Stenotrophomonas maltophilia Strain B418, a Promising Agent for Biocontrol of Plant Pathogens and Root-Knot Nematode

**DOI:** 10.1128/genomeA.00015-15

**Published:** 2015-02-19

**Authors:** Yuanzheng Wu, Yilian Wang, Jishun Li, Jindong Hu, Kai Chen, Yanli Wei, Dmitry P. Bazhanov, Alesia A. Bazhanova, Hetong Yang

**Affiliations:** Biotechnology Center of Shandong Academy of Sciences, Shandong Provincial Key Laboratory of Applied Microbiology, Shandong Province, Jinan, China

## Abstract

Stenotrophomonas maltophilia strain B418 was isolated from a barley rhizosphere in China. This bacterium exhibits broad-spectrum inhibitory activities against plant pathogens and root-knot nematode along with growth-promoting effects. Here, we present the draft genome sequence of S. maltophilia B418.

## GENOME ANNOUNCEMENT

Stenotrophomonas maltophilia strain B418, isolated from the barley rhizosphere in Shandong Province (1), is a multifunctional plant growth-promoting rhizobacterium (PGPR) capable of colonizing wheat roots and stimulating their elongation through the solubilization of phosphate and potassium (2). Initial phenotypic characteristics placed B418 as Burkholderia vietnamiensis, until further studies revealed it as a member of S. maltophilia. Although S. maltophilia is well known as a nonfermentative Gram-negative bacterium causing nosocomial infections (3, 4), many strains of this species are identified as beneficial bacteria with the ability to produce antibiotics and plant growth-promoting factors and have long been used for biocontrol in agriculture (5–7). The strain B418 could also suppress cucumber seedling blight caused by *Rhizoctonia* *solani* and wheat sheath blight caused by R. cerealis (8), as well as inhibit eggplant root-knot nematode disease by Meloidogyne incognita (9). Here, we sequenced the genome of B418 to gain insight into its genetic and physiological properties that may contribute to plant health.

The whole-genome shotgun sequencing of S. maltophilia B418 was performed using the Illumina HiSeq2000 platform with a Solexa paired-end (PE) sequencing system and PCR-based gap filling strategy. Two sets of Illumina PE libraries with insert sizes of 180 and 500 bp were constructed and sequenced. The sequencing yielded a total of 975,986,100 and 853,386,300 bases, together with 3,253,287 and 2,844,621 paired-end reads, respectively. The resulting clean reads were *de novo* assembled into 231 large contigs (>500 bp) using Velvet version 1.2.03 (10), and 157 scaffolds were obtained using SSPACE basic version 2.0 (11). The protein-coding sequences (CDSs) were identified with Glimmer 3 (12), while tRNA and rRNA genes were predicted by tRNAscan-SE (13) and RNAmmer (14), respectively. The draft genome of *S. maltophilia* B418 is characterized as a circular chromosome of 4,712,807 bp with a 65.36% G+C content without plasmids. The chromosome harbors 3,956 CDSs, 39 tRNA genes, and 5 rRNA operons.

Genome analysis revealed that the genome of S. maltophilia B418 contains various gene clusters for biosynthesis of secondary metabolites and antimicrobial peptides such as enterobactin, phenazine, and pyrrolnitrin (15, 16). The genome information displays several antibiotic resistance genes encoding SmQnr, efflux pumps, and bleomycin resistance protein (17). The presence of aspartic proteinase, serine proteinase, and chitinase genes confirms its ability to inhibit the growth of plant pathogens and root-knot nematode (18). There also exist in the genome multiple genes encoding acid phosphatase, alkaline phosphatase, and phosphotase, which may be involved in phosphate solubilization (19). Besides these genes, a large number of epiphytic fitness genes are identified, including those associated with pili, flagella, and adhesions, which can contribute to the rhizosphere competence for approaching environmental nutrients (20). Overall, the genome sequence of B418 provides better avenues for exploiting the biocontrol mechanism possessed by S. maltophilia.

### Nucleotide sequence accession numbers.

This whole-genome shotgun project has been deposited at DDBJ/EMBL/GenBank under the accession number JSXG00000000. The version described in this paper is version JSXG01000000.
